# Mutations in *CHMP2B* in Lower Motor Neuron Predominant Amyotrophic Lateral Sclerosis (ALS)

**DOI:** 10.1371/journal.pone.0009872

**Published:** 2010-03-24

**Authors:** Laura E. Cox, Laura Ferraiuolo, Emily F. Goodall, Paul R. Heath, Adrian Higginbottom, Heather Mortiboys, Hannah C. Hollinger, Judith A. Hartley, Alice Brockington, Christine E. Burness, Karen E. Morrison, Stephen B. Wharton, Andrew J. Grierson, Paul G. Ince, Janine Kirby, Pamela J. Shaw

**Affiliations:** 1 Department of Neuroscience, University of Sheffield, Sheffield, South Yorkshire, United Kingdom; 2 Department of Neurology, University of Birmingham, Birmingham, East Midlands, United Kingdom; National Institutes of Health, United States of America

## Abstract

**Background:**

Amyotrophic lateral sclerosis (ALS), a common late-onset neurodegenerative disease, is associated with fronto-temporal dementia (FTD) in 3–10% of patients. A mutation in *CHMP2B* was recently identified in a Danish pedigree with autosomal dominant FTD. Subsequently, two unrelated patients with familial ALS, one of whom also showed features of FTD, were shown to carry missense mutations in *CHMP2B*. The initial aim of this study was to determine whether mutations in *CHMP2B* contribute more broadly to ALS pathogenesis.

**Methodology/Principal Findings:**

Sequencing of *CHMP2B* in 433 ALS cases from the North of England identified 4 cases carrying 3 missense mutations, including one novel mutation, p.Thr104Asn, none of which were present in 500 neurologically normal controls. Analysis of clinical and neuropathological data of these 4 cases showed a phenotype consistent with the lower motor neuron predominant (progressive muscular atrophy (PMA)) variant of ALS. Only one had a recognised family history of ALS and none had clinically apparent dementia. Microarray analysis of motor neurons from *CHMP2B* cases, compared to controls, showed a distinct gene expression signature with significant differential expression predicting disassembly of cell structure; increased calcium concentration in the ER lumen; decrease in the availability of ATP; down-regulation of the classical and p38 MAPK signalling pathways, reduction in autophagy initiation and a global repression of translation. Transfection of mutant *CHMP2B* into HEK-293 and COS-7 cells resulted in the formation of large cytoplasmic vacuoles, aberrant lysosomal localisation demonstrated by CD63 staining and impairment of autophagy indicated by increased levels of LC3-II protein. These changes were absent in control cells transfected with wild-type CHMP2B.

**Conclusions/Significance:**

We conclude that in a population drawn from North of England pathogenic *CHMP2B* mutations are found in approximately 1% of cases of ALS and 10% of those with lower motor neuron predominant ALS.

We provide a body of evidence indicating the likely pathogenicity of the reported gene alterations. However, absolute confirmation of pathogenicity requires further evidence, including documentation of familial transmission in ALS pedigrees which might be most fruitfully explored in cases with a LMN predominant phenotype.

## Introduction

Amyotrophic lateral sclerosis (ALS) is a late-onset, relentlessly progressive and eventually fatal neurodegenerative disorder characterised by the injury and death of upper motor neurons (UMN) in the cortex and lower motor neurons (LMN) in the brainstem and spinal cord [1]. The disorder comprises a range of clinical phenotypes depending on the pathoanatomical distribution of the motor system degeneration. Classical ALS is a combined UMN and LMN disorder. The pure LMN disorder of progressive muscular atrophy (PMA) and the pure UMN disorder of primary lateral sclerosis (PLS) share common molecular pathology hallmarks with ALS, and are considered syndromic variants. The majority of ALS cases are sporadic, although 5–10% of cases are familial. Fifteen loci are known to be associated with ALS, and eight causative genes have been identified, the most common of which is *SOD1* (*Cu/Zn superoxide dismutase 1*) [2]. Recently, we identified missense mutations in *CHMP2B* (charged multivesicular protein 2B) in two individuals with familial ALS, one of whom had associated features of frontotemporal dementia (FTD) [3]. *CHMP2B* is expressed in all major areas of the human brain, as well as multiple other tissues outside the CNS. Although the exact function of CHMP2B is unknown, its yeast orthologue, vacuolar protein sorting 2 (VPS2), is a component of the ESCRTIII complex (endosomal secretory complex required for transport) [4]. ESCRTIII is an important component of the multivesicular bodies (MVBs) sorting pathway, which plays a critical role in the trafficking of proteins between the plasma membrane, trans-Golgi network and vacuoles/lysosome [5]. Alterations to *VPS2* in yeast results in the formation of dysmorphic hybrid vacuole-endosome structures; additionally, disruption to other ESCRTIII components abolishes the ability of MVBs to internalise membrane-bound cargoes [5], [6], [7]. Interestingly, two other MND-causing genes, *ALS2* and *ALS8*, are proposed to contribute to motor neuron injury by causing disruption to the processes of endocytosis and vesicle trafficking [8], [9], [10], [11].

Defects in *CHMP2B* were originally reported in a Danish pedigree with autosomal dominant FTD [4]. The G>C single nucleotide change in the acceptor splice site of exon 6 of *CHMP2B* affected mRNA splicing, resulting in two aberrant transcripts: inclusion of the 201-bp intronic sequence between exons 5 and 6 (*CHMP2B^Intron5^*), or a short deletion of 10bp from the 5′ end of exon 6 (*CHMP2B^Δ10^*). Expression of mutant CHMP2B protein in cells resulted in aberrant structures dispersed throughout the cytosol [4], and the ectopic expression of *CHMP2B^Intron5^* in cortical neurons caused dendritic retraction prior to neurodegeneration [12]. In addition, autophagosome accumulation and the inhibition of autophagy have been seen in cells expressing the CHMP2B mutations found in FTD [12], [13].

Several neurodegenerative diseases are now believed to contain an element of dysregulation of the lysosomal degradation of proteins, as reviewed by Martinez-Vicente *et al.* [14]. The results from these recent studies are part of a growing body of evidence that common pathways are involved in a spectrum of neurodegenerative diseases [15]. It was recently discovered that the ubiquitin positive inclusions seen in FTD and ALS contain the same protein, TAR DNA-binding protein-43 (TDP43), and that mutations in progranulin (*PGRN*) give rise to FTD with ubiquitin-positive inclusion bodies similar to those seen in some ALS patients [16], [17]. It is proposed that mutations in *CHMP2B* may lead to FTD and other neurodegenerative diseases, including ALS, which is predicted to involve disruption to the cellular processes involved in the recycling and degradation of proteins. However, the status of *CHMP2B* mutations as a contributor to ALS has remained uncertain, due to the lack of described pedigrees where mutations segregate with disease in multiple affected individuals. The aims of this study were i) to identify whether mutations in *CHMP2B* contribute significantly to the pathogenesis of familial and apparently sporadic ALS, by screening for genetic alterations in a cohort of 433 ALS cases in whom the clinical phenotype had been documented serially throughout the disease course; ii) to investigate in CNS tissue changes in the gene expression profile of motor neurons from cases with *CHMP2B* mutations compared to neurologically normal controls, and iii) to investigate the functional effects of *CHMP2B* mutations in an *in vitro* system. We propose that *CHMP2B* missense mutations associate with ALS and demonstrate that these mutations give rise to a distinct clinical and neuropathological phenotype, cause a distinctive alteration in the motor neuron transcriptome and disrupt cellular pathology, compared to controls.

## Methods

### Patients and controls

DNA samples from 433 ALS cases were screened. Of these 37 had familial ALS (FALS) and were negative for mutations in *SOD1*, *TDP43*, *FUS/TLS*, *ANG* and *VAPB*; 356 had classical sporadic ALS with UMN and LMN clinical signs and 40 had ALS with a LMN phenotype throughout the disease course (PMA variant). Autopsy CNS tissue was available for 123 of the cases screened and for all the patients in whom a mutation in CHMP2B was found. DNA control samples (N = 500) were obtained from the Sheffield and Birmingham MND DNA banks and the Newcastle Brain Tissue Resource. All control individuals were neurologically normal and matched to the disease cohort by age and sex. Approval for the use of DNA samples was obtained from the South Sheffield Research Ethics Committee and written consent was obtained from the donors. Ethnicity of cases and controls was UK Caucasian.

### PCR amplification and mutation screening of CHMP2B

DNA was extracted from snap frozen cerebellum or blood as described previously [18]. PCR was performed as described in the original *CHMP2B* publication [4]. Following clean-up with ExoSAP-IT (GE Healthcare, UK), PCR products were bidirectionally sequenced [18]. The *CHMP2B* nucleotide sequence was taken from ENST00000263780 on the Ensembl database, and was used to determine sites of known polymorphisms. Nucleotides were numbered in accordance with the nomenclature recommended by the Human Genome Variation Society (www.hgvs.org).

To determine the prevalence of the c.311C>A change in the control population, exon 3 PCR products were digested with the restriction enzyme *Acc*I, generating 244, 104 & 51bp fragments from the wild type sequence. The C to A substitution abolishes a restriction site, resulting in two fragments (244 & 155bp). The c.618A>C change was screened by digestion of exon 6 PCR products with *Ban*I, which generated 207 & 96bp in the presence of the substitution, whilst the wild type sequence of 303bp remained uncut. The presence of c.-151C>A and c.85A>G in the control population were determined by bidirectional sequencing of exons 1 and 2, respectively.

### Neuropathology

Brains and spinal cords were dissected so that one cerebral hemisphere, the midbrain, left hemi-brainstem and left cerebellar hemisphere were sliced for snap freezing. Selected spinal cord segments were also snap frozen. The remaining tissues were fixed in formalin for processing to paraffin wax and used in routine staining and immunocytochemistry from all CNS levels. Standard immunocytochemical methods, including antigen retrieval where appropriate, were used to demonstrate localization of ubiquitin, p62/sequestosome 1, TDP43, CD68 (a marker of microglial activation), α-synuclein and AT8 (a tau marker) (Table 1). The pathological survey reported here includes examination of cervical and lumbar limb enlargements of the spinal cord, thoracic spinal cord, multiple medulla oblongata and pontine levels to include lower cranial motor nerve nuclei, upper pons and midbrain, the hippocampus, motor cortex, frontal and temporal neocortex and cerebellum.

**Table 1:** Antibody source and conditions.

Antibody (clone)	Isotype	Dilution	Antigen retrieval	Source
CD68 (PG-M1)	IgG3	1∶200	microwave 10min citrate buffer	DAKO
Ubiquitin	poly	1∶200	microwave 10min citrate buffer	DAKO
Tau (AT8)	IgG_1K_		microwave 10min citrate buffer	Pierce Endogen
α-synuclein	IgG	1∶200	microwave 10min citrate buffer	Zymed
p62/sequestosome	poly	1∶200	microwave 10min citrate buffer	Progen

### Microarray analysis of cervical motor neurons from CHMP2B cases vs. controls

Snap frozen cervical spinal cord was available for cases 1–3, but not case 4, and the cervical cord from 7 control cases was used for comparison. Ten micron sections were prepared, and 500 motor neurons were isolated from each sample using laser-capture microdissection (LCM); RNA was extracted as previously described [19]. The quality (2100 bioanalyzer, RNA 6000 Pico LabChip; Agilent, CA, USA) and quantity (NanoDrop 1000 spectrophotometer) of the RNA from all of the samples were assessed. Each RNA sample was linearly amplified, following the Eberwine procedure [20] using the Two-cycle Amplification Method (Affymetrix), and again checked for quality and quantity. Fifteen micrograms of amplified cRNA from the 3 mutant *CHMP2B* cases and 7 controls was fragmented and each hybridised individually to 10 Human Genome U133 Plus 2.0 GeneChips (Affymetrix), as per manufacturer's protocols. Following stringency washes, chips were stained and scanned, and GeneChip Operating Software (GCOS) used to produce signal intensities for each transcript. ArrayAssist (Iobion Informatics, CA, USA) was used to determine genes that showed significant differential expression in the presence of *CHMP2B* mutations compared to control samples. Transcripts were considered differentially expressed if there was a twofold or greater difference in the mean signal intensity of the *CHMP2B* cases compared to the control group (p<0.05, two-tailed *t* test). Transcripts in which the signal intensities on all 10 GeneChips were below 35 (the average intensity of background noise level) were discarded as were those transcripts of unknown function. The differentially expressed transcripts on the resulting list were classified by biological process, as determined by GeneOntology terms. To identify specific pathways affected by *CHMP2B* mutations, PathwayArchitect (Stratagene) and the DAVID Functional Annotation Tool bioinformatics software packages were used [21].

### Validation of microarray results by Q-PCR

Primers were designed and optimised to validate significant changes in expression of genes with key roles in the pathways affected by *CHMP2B* mutations (Table 2). Q-PCR was perfomed using 25ng of cDNA, 1× Brilliant II SYBR Green QPCR Master Mix (Agilent, CA, USA), the optimised concentrations of forward and reverse primers, and nuclease free water was used to make a final reaction volume of 20µl. Samples were run on an Mx3000P Real-Time PCR system (Stratagene) using previously published parameters [19]. Gene expression values, normalised to actin expression, were determined using the ddCt calculation [22]. Actin was selected as a housekeeping gene as its expression was consistent across the 10 GeneChips. An unpaired two-tailed *t* test was used to analyse the data and to determine the statistical significance of any differences in gene expression (GraphPad Prism 5, Hearne Scientific Software).

**Table 2:** Primer sequences for Q-PCR validation of selected genes.

Gene name	Primer name	Primer sequence	Concentration (nM)
Tubulin, beta	TUBB-F	5′-GTCACCTTCATTGGCAATAGCA-3′	900
	TUBB-R	5′-GCGGAACATGGCAGTGAACT-3′	600
Microtubule-associated protein 4	MAP4-F	5′-GGACCAGCTTTCCTCCGTAGA-3′	900
	MAP4R	5′-GACTACGCAACCCTGTTTCCTT-3	600
Autophagy gene 1	ATG1-F	5′-CGCCACATAACAGACAAAAATACAC-3′	900
	ATG1-R	5′-CCCCACAAGGTGAGAATAAAGC-3′	900
Kinesin family member 1A	KIF1A-F	5′-GAGAGTCTGGTCATAGGAGTCATGTC-3′	600
	KIF1A-R	5′-GGCTACTGTCTTTCCTTGAGCTAAA-3′	600
Na+/Ca2+ exchanger	NCX1-F	5′-TTATAGAGACGTTGATATGTTGGATGTG-3′	600
	NCX1-R	5′-ACAGTGCAGATGTGAAATAAATACTTTG-3′	300
Sarcoplasmic/endoplasmic reticulum calcium ATPase 2	SERCA-F	5′-TGGAGTAACCGCTTCCTAAACC-3′	900
	SERCA-R	5′-TACTTTTCTTTTTCCCCAACATCAG-3′	900
E2F Transcription factor 6	E2F6-F	5′-GCGGAAAAGTCTGAGCTGTGTAGT-3′	600
	E2F2-R	5′-GACCTCTCCTACTCTTGTGGCTTAA-3′	600
Cadherin 13	CDH13-F	5′-GCCAAGAAAAGGGCTGACATT-3′	600
	CDH13-R	5′-GTGTCCCCATTAGAATCAGTACGA-3′	600

### *In vitro* models to investigate the functional effects of CHMP2B mutations

HEK-293 cells (ECACC) were plated on 13mm coverslips in Dulbecco's Modified Eagle's Medium ((DMEM) w Glucose + L-glutamine, w/o Na pyruvate; Lonza) plus 10% fetal calf serum (FCS, Biosera), in the absence of antibiotic (termed +/−). Cells were transfected with 500ng myc-tagged *CHMP2B* with either the wild-type, c.85A>G (p.I29V), c.311C>A (p.T104N) or c.618A>C (p.Q206H) sequence, under a constitutive cytomegalovirus promoter, using Lipofectamine 2000 (Invitrogen) as per the manufacturer's protocol. After transfection, cells were grown in DMEM plus 10% FCS for 24 hours. To investigate changes in the lysosomal degradation pathway, autophagy was induced in selected cells by serum withdrawal for two hours, 22 hours post-transfection. Cells are subsequently referred to as +/− (10% FCS, no antibiotic) or −/− (no FCS for the last 2 hours of growth post-transfection, no antibiotic). Cells were fixed, (4% paraformaldehyde (Sigma)), permeabilised (0.1% triton (Sigma)), and blocked in 5% goat serum (Sigma) in PBS for one hour. Primary antibodies were mouse α-CD63 (in-house), a marker of late-endosomes and lysosomes, and either rabbit or mouse α-myc (AbCam). Secondary antibodies were anti-mouse or α-rabbit AlexaFluor488 and α-mouse or α-rabbit AlexaFluor555 (Molecular Probes). Cells were visualised using the Zeiss Axioplan2 microscope and the Zeiss LSM 510 confocal microscope. Transfected cells were scored for the presence of vacuoles and halos. To measure vacuole area, images were captured from a minimum of 25 fields of view per transfection round. ImageJ was used to convert the images to greyscale, subtract the background and adjust the threshold. The ‘analyse particles’ plug-in was used to measure the area of the vacuoles in µm^2^.

COS-7 cells (ECACC) were plated in 6 well tissue culture plates in DMEM, plus 10% FCS, in the absence of antibiotic 24 hours prior to transfection (DMEM+/−). Cells were transfected with 2µg of plasmid DNA, as described above, using Lipofectamine 2000 as per the manufacturer's protocol. To induce autophagy, 22 hours post-transfection cells were serum starved for 2 hours, before being washed in PBS and collected in 500µl 1× Trypsin-EDTA (TE). An equal volume of DMEM+/− was added to the cells to quench the TE, and cells were pelleted by spinning at 3,000×*g* for 2 minutes. Cells were washed with 500µl PBS and spun again to re-pellet. The supernatant was discarded and cells were resuspended in 40µl of lysis buffer (25µM Tris pH7.4, 0.5% (v/v) Triton, 50mM NaCl, 2mM EDTA, plus protease inhibitor cocktail) and lysed at 4°C. Protein concentration was estimated using a Bradford assay, and 20µg of total protein per sample was separated by sodium dodecyl sulfate polyacrylamide gel electrophoresis (SDS-PAGE) (12% acrylamide gels) and transferred to PVDF membranes. Blots were blocked in TBS-T (20mM Tris-HCl pH7.6, 137mM NaCl, plus 0.1% (v/v) Tween-20) and 5% (w/v) dried skimmed milk. They were then probed with rabbit polyclonal anti-LC3 (Stratech) diluted 1∶1000 and rabbit polyclonal anti-actin diluted 1∶1000 (used as a protein loading control) in TBS-T plus milk for one hour at room temperature, followed by peroxidase-conjugated secondary antibody (1∶4000, one hour at room temperature). Antibody binding was revealed using enhanced chemiluminescence, as per the manufacturer's instructions.

## Results

### Mutation screening of CHMP2B in ALS patients

**Figure 1:**
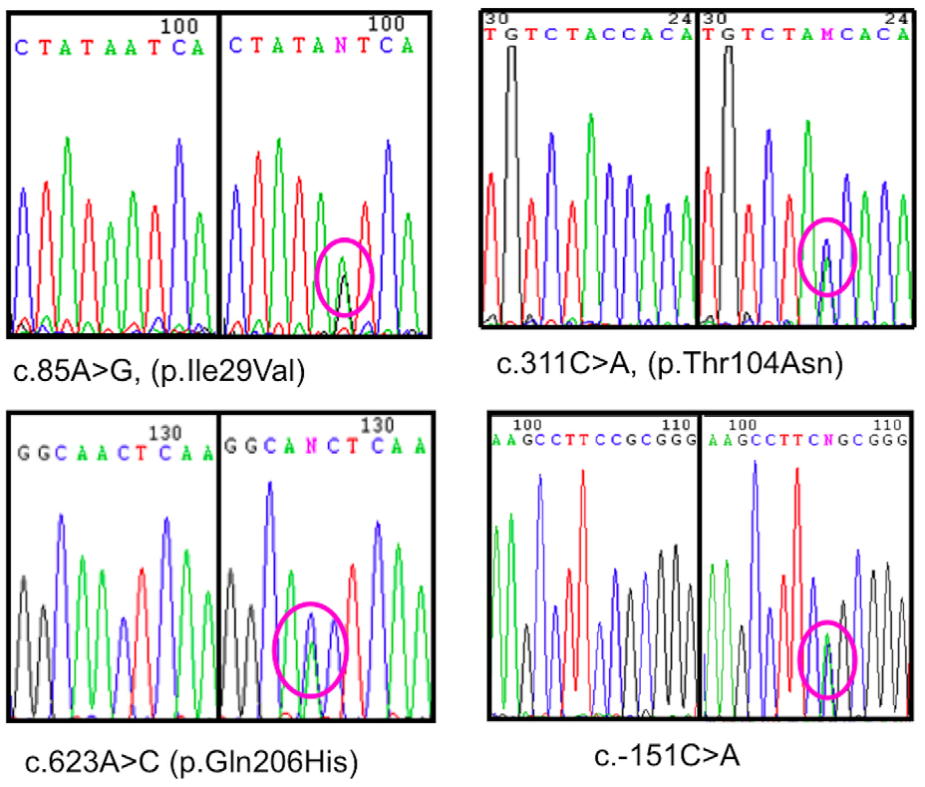
Chromatograms showing nucleotide changes in *CHMP2B*. On the left side of each image is the normal wild-type sequence, whilst the right side shows the nucleotide change for each of the changes identified in *CHMP2B*.

Sequence analysis of the entire coding region and intron/exon boundaries of *CHMP2B* from 433 ALS cases identified point mutations in four cases (0.9%) (Figure 1). One patient (Case 1) was heterozygous for a previously undescribed mutation, a single nucleotide substitution, c.311C>A, which results in the substitution of threonine by asparagine (p.T104N). Two subjects (Cases 2 & 3), were heterozygous for a c.85A>G substitution, resulting in a previously described isoleucine to valine substitution (p.I29V). The fourth patient (Case 4) was the previously published glutamine to histidine (c.618A>C, p.Q206H) case [3]. These mutations are all highly conserved in mammals, with the p.T104N and p.Q206H also conserved in chicken and zebrafish (see Figure S1). The c.311C>A, c.85A>G and c.618A>C changes were absent in 1000 control chromosomes from 500 neurologically normal individuals. The c.618A>C mutation has previously been shown to be absent in 1280 control chromosomes [3]. A novel SNP, c.-151C>A, was also identified in the 5′UTR of *CHMP2B* in four cases (0.9%), however, this change was also present in 2% of controls.

### Clinical phenotypes of patients carrying mutations in CHMP2B

In all cases neurological investigations including haematological and biochemical blood tests, CSF analysis, neuroimaging and neurophysiological evaluation were compatible with a diagnosis of ALS. None of the cases presented with or developed signs of frontotemporal dementia (FTD).

Case 1 was a 54 year old man who presented with respiratory failure and bulbar dysfunction. His first problem was dyspnoea whilst swimming. He was treated with non-invasive ventilation (NIV) from the time of diagnosis throughout his illness. There was no reported family history of neurological disease. On examination at the time of presentation, he had a moderately severe dysarthria and bilateral wasting, fasciculation and weakness of the tongue. He had wasting and proximal fasciculation in the upper and lower limbs, weakness of the ulnar and median innervated intrinsic hand muscles and the hip flexors. The deep tendon reflexes were normal and plantar responses were flexor, consistent with a pure lower motor neuron phenotype. His symptoms progressed rapidly over the course of 15 months, at which stage he died of respiratory failure with relatively preserved limb function.

Case 2 was a 64 year old female who presented with back pain and progressive weakness of the right leg. She underwent L4/5 spinal decompression to no avail and her symptoms continued to progress to affect both legs, with later development of upper limb, bulbar and respiratory muscle weakness. There was no significant family history. On examination she had wasting and predominantly distal weakness in the lower limbs with upper limb fasciculations. The reflexes were normal throughout, and the plantar reflexes were flexor. She died 66 months after symptom onset.

Case 3 was a 49 year old man who initially noticed a deterioration in his ability to play football, due to weakness affecting his left leg. His symptoms progressed steadily. Fifteen months after onset he had wasting and fasciculations in the upper limbs with preserved power. The lower limbs were wasted globally, most severely in the left anterior tibial compartment. Power in hip flexion and ankle dorsiflexion was reduced on the left and the ankle jerks were absent bilaterally. He developed bulbar symptoms 24 months after disease onset. No UMN signs were detectable clinically throughout the disease course. There was no family history of ALS, with healthy parents and 5 siblings, including one with developmental delay described as possible autism. He died of respiratory failure 29 months after his initial symptom onset.

Case 4 was a 73 year old male who presented with dysarthria, dysphagia and clumsiness of the hands. His symptoms progressed rapidly and 12 months after onset he had a wasted, fasciculating tongue, LMN weakness in the upper and lower limbs, depressed reflexes and flexor plantar responses. He died 14 months after disease onset. He had a cousin who had died from ALS several years earlier.

### Neuropathological findings in the four cases with CHMP2B mutations

**Figure 2:**
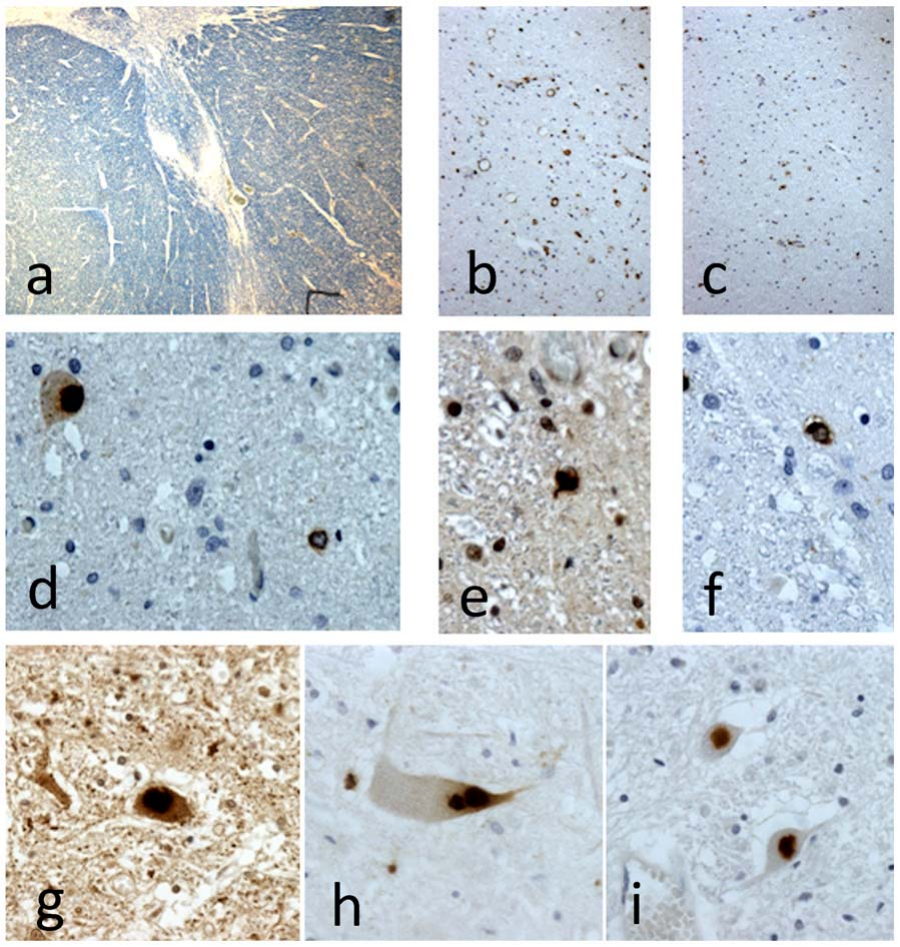
Photomicrography of pathological changes associated with CHMP2B mutations. In all cases there was no evidence of myelin pallor affecting the corticospinal tracts (**a**). Case 1 showed some minor upregulation of CD68 immunoreactivity in the spinal lateral corticospinal tracts (**b**) compared with the dorsal columns (**c**). Sequestosome 1/p62 staining showed compact intraneuronal inclusions in spinal motor neurons and occasional glial inclusions (**d**). The glial inclusions show coiled body morphology immunoreactive for both TDP43 (**e**) and p62 (**f**). All cases showed a predominance of compact intraneuronal inclusions in motor neurons (**g–i**). (a: Luxol fast blue; b,c: CD68; d,f,h,i: p62; e,g: TDP43. Microscopy at ×2 obj. (a); ×10 obj. (b,c); ×40 obj. (d–i)).

The anatomical and molecular pathology of all four cases was similar and corresponded to a rather stereotypical pattern of LMN predominant degeneration (Figure 2). Involvement of UMNs was not detectable in conventional stains and the Betz cell somata were readily identified and appeared normal in all cases. There was no evidence of myelin pallor in the corticospinal tracts (Figure 2a) other than equivocal changes in the medullary pyramid of one case (Case 1), which was not present in the spinal cord at any level. Microglial activation demonstrated by CD68 staining is a more sensitive indication of early white matter tract degeneration [23]. Three of the cases showed no evidence for corticospinal tract degeneration at any level. One case (Case 1) showed marked subcortical microglial activation centred on the precentral gyrus, mild changes in the medulla and only equivocal changes in the spinal cord white matter. Glial pathology was previously reported in the motor cortex in case 4, comprising oligodendroglial coiled bodies immunoreactive for p62 and TDP43, which were not readily demonstrated by conventional ubiquitin immunocytochemistry [3]. These lesions were present in the motor cortex and the ventral horns of all the cases. These lesions are not confined to *CHMP2B*-related ALS as previously reported [3], but are consistently present to a variable degree in sporadic and non-SOD1-related familial variants of ALS [24].

The LMN pathology in all cases was typical of the primary muscular atrophy (PMA) variant of ALS. There was variable severe loss of motor neurons from all spinal levels. Bulbar motor nuclei were generally less severely affected. Surviving motor neurons contained a population of inclusion bodies demonstrated by ubiquitin, p62 and TDP43 staining. In three cases these were exclusively of ‘compact’ morphology with no demonstrable ‘skeins’ (Figures 2d,g,h, i). All the glia and neurones containing TDP43 intracytoplasmic inclusions showed decreased staining in the cell nucleus in comparison to retention of normal nuclear staining in cells negative for inclusions. One case showed very few intraneuronal lesions, in the context of massive motor neuronal loss, of rather indeterminate morphology. Bunina bodies were absent from all the cases. Extramotor involvement of the CNS assessed by p62/TDP43 immunoreactive lesions was absent including evaluation of the hippocampal dentate granule cells and the frontal and temporal neocortex. Staining for related neurodegenerative pathologies (tau and alpha-synuclein) showed minimal expression of Alzheimer's disease pathology (low Braak stage and minimal cortical amyloid deposition) and absence of synucleinopathy.

### Gene expression profiling of motor neurons from CHMP2B cases compared to controls

The percentage of genes described as present on the 10 GeneChips was an average of 22.7% for the three available *CHMP2B* cases (2× p.I29V, 1× p.T104N), and 28.2% for the seven neurologically normal control cases. (CEL files for each of the 10 GeneChips have been submitted to the Gene Expression Omnibus Repository, Accession GSE19332). ArrayAssist (Affymetrix) was used to analyse expression data. Duplicate probes were removed, as were probes for unknown genes, and those for which the signal intensity in all 10 GeneChips was below 35 (the average background level). After filtering, 890 genes were found to be downregulated, and 555 upregulated, all with a fold change ≥2, and p value ≤0.05. These 1,445 differentially expressed genes were then categorised according to their biological process, as defined by GeneOntology terms (Table 3) (Full list available in Table S1). An additional 398 genes were downregulated and 358 upregulated at a fold change of 1.5–1.99, p≤0.05. These were only considered if they were involved in the pathways of interest.

**Table 3:** Grouping of 891 downregulated and 556 upregulated genes, with a fold change (FC) ≥2, and p value ≤0.05, into their biological process, as defined by GeneOntology terms.

	Number of genes FC ≥2, p≤0.05	Number of genes FC ≥2, p≤0.05
Biological process	Downregulated	Upregulated
Apoptosis	16	9
Cell adhesion	34	16
Cell cycle	49	24
Cell motility	10	1
Cytoskeleton	23	17
Development	8	13
Immune response	18	29
Ion transport	20	29
Kinases/phosphatases	19	8
Metabolism	88	36
Protein cleavage/degradation	41	32
Protein folding	5	2
Protein modification	25	15
RNA processing	32	19
Signalling	55	64
Stress response	13	6
Transcription	84	76
Translation	56	7
Transport	77	35
Miscellaneous	101	49
Unknown	118	69

The functional annotation tool of the DAVID Bioinformatics Resource was used to identify pathways with a significant number of differentially expressed genes, namely: axon guidance, regulation of actin cytoskeleton and SNARE interactions in vesicular transport, mammalian target of rapamycin (mTOR) signalling and regulation of autophagy, mitogen activated kinase (MAPK) signalling, calcium signalling, and cell cycle and apoptosis (Table 4). We focused our analysis on pathways that were of most biological interest in relation to the predicted function of CHMP2B and related proteins.

**Table 4:** Genes altered in calcium signalling, MAPK signalling, axon guidance, cell cycle and apoptosis, regulation of actin cytoskeleton and SNARE interactions in vesicular transport, and mTOR signalling and regulation of autophagy in *CHMP2B* motor neurons compared to neurologically normal controls.

Pathway/Probe ID	Gene symbol	Fold change
***Calcium signalling pathway***		
1559633_a_at	CHRM3	2.92
211426_x_at	Gq	−3.08
204248_at	Gq11	−2.51
240052_at	IP3R	−1.93
235518_at	NCX	3.21
212826_s_at	ANT3	−1.82
208844_at	VDAC3	2.93
216033_s_at	FYR	−2.88
209186_at	SERCA	2.12
***MAPK signalling pathway***		
1560689_s_at	AKT	1.87
1552264_a_at	ERK2	−1.71
201841_s_at	HSP27	−12.85
215050_x_at	MK2	−1.6
223199_at	MNK2	−3.24
210631_at	NF1	1.81
211561_x_at	p38	−5.24
224411_at	PLA2G12B	2.58
233470_at	PTPN5	1.59
201244_s_at	RAF1	−2.1
212647_at	RAS	−4.96
217714_x_at	STMN1	1.6
211537_x_at	TAK1	−2.42
***Axon guidance***		
1562240_at	PLXNA4A	2.32
229026_at	CDC42SE2	3.38
235412_at	ARHGEF7	4.37
208009_s_at	ARHGEF16	2
226576_at	ARHGAP26	−2.39
230803_s_at	ARHGAP24	−3.9
212647_at	RRAS	−4.96
206281_at	ADCYAP1	−9.4
217480_x_at	NTN2L	2.67
210083_at	SEMA7A	1.82
211651_s_at	LAMB1	−3.81
216840_s_at	LAMA2	−3.86
203071_at	SEMA3B	−5.39
216837_at	EPHA5	2.35
206070_s_at	EPHA3	−3.31
***Cell cycle & apoptosis***		
212312_at	BCL2L1	−2.37
208876_s_at	PAK2	2.92
209364_at	BAD	−3.92
228361_at	E2F2	2.73
231237_x_at	E2F5	6.93
203957_at	E2F6	2.8
229468_at	CDK3	1.89
1561190_at	CDKL3	3.14
208656_s_at	CCNI	−2.03
231198_at	CDK6	−2.15
205899_at	CCNA1	−2.24
1555411_a_at	CCNL1	−3.29
208711_s_at	CCND1	−5.88
212983_at	HRAS	−2.71
201244_s_at	RAF1	−2.1
211561_x_at	p38	−5.24
1552264_a_at	ERK1	−1.71
201202_at	PCNA	−2.68
207574_s_at	GADD45B	−2.44
***Regulation of actin cytoskeleton & SNARE interactions in vesicular transport***		
209244_s_at	KIF1C	−2.22
203129_s_at	KIF5C	−2.8
203849_s_at	KIF1A	−3.02
217917_s_at	DYNLRB1	−2.8
229106_at	DYNLL2	−2.99
229115_at	DYNC1H1	−3.54
218522_s_at	MAP1S	−2.83
212567_s_at	MAP4	−3.1
217714_x_at	STMN1	1.6
212639_x_at	TUBA1B	−3.98
216323_x_at	TUBA3D	−3.33
210527_x_at	TUBA3C	−3.55
213646_x_at	TUBA1B	−4.48
211750_x_at	TUBA1C	−3.94
202154_x_at	TUBB3	−4.13
209026_x_at	TUBB	−4.87
208977_x_at	TUBB2C	−5.18
***mTOR signalling and regulation of autophagy***		
200709_at	FKBP12	−1.79
209333_at	ATG1	−3.48
201254_x_at	RPS6	−2.28
216105_x_at	PP2A	−2.03
211787_s_at	EIF4A1	−3.04
211937_at	EIF4B	−1.93
200596_s_at	EIF3A	−2.03
203462_x_at	EIF3B	−3.97
200647_x_at	EIF3C	−3.3
200005_at	EIF3D	−2.28
208887_at	EIF3G	−1.8
208756_at	EIF3I	−5.79

(Fold change: positive numbers indicate upregulated, whilst negative numbers depict downregulated transcripts).

#### Regulation of actin cytoskeleton and SNARE interactions in vesicular transport (Figure 3)

**Figure 3:**
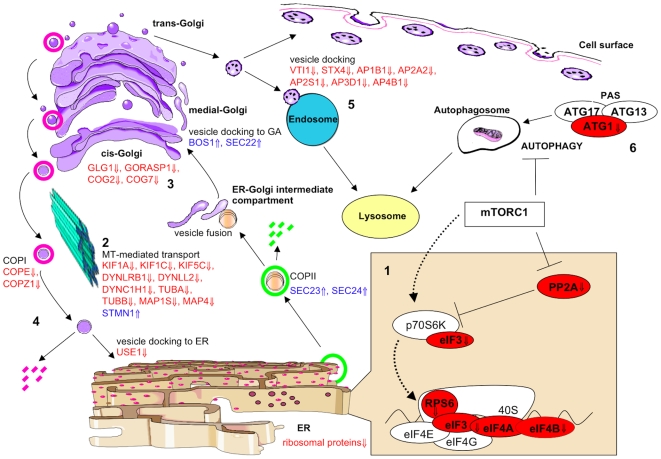
Summary of key gene expression changes to ER and Golgi function, vesicular transport, mTOR signalling and autophagy. The downregulation of multiple transcripts encoding ribosomal proteins, translation initiation factors (eIFs) and the ribosomal subunit, RPS6 suggests a global repression of translation within the cell (1). SEC23 and SEC24 coat vesicles into which immature proteins are packaged and are upregulated. Vesicles are transported along microtubules (MTs) from the ER-Golgi intermediate compartment; however, downregulation of the main components of microtubules, TUBA and TUBB, and the MT-stabilising proteins, MAP1S and MAP4, indicates MT disassembly and therefore disruption to vesicular transport (2). This is enhanced by upregulation of STMN1, a MT-destabilising protein, whose over-expression has also been demonstrated to result in Golgi fragmentation. Downregulation of transcripts maintaining Golgi structure (GLG1, GORASP1, COG2 and COG7) support the hypothesis of Golgi fragmentation (3). COPI is used to coat empty vesicles exiting the Golgi for recycling back to the ER, however, two main constituents, COPE and COPZ1, are downregulated, as is USE1, which is required for vesicle fusion with the ER. These findings predict an eventual deficit of material available to ER for the packaging of newly synthesised proteins (4). There is dysregulation of multiple SNARE transcripts, which are required for vesicle fusion. BOS1 and SEC22 are upregulated, which may be the Golgi's response to the reduction in vesicles being transported along destabilised microtubules. Multiple SNAREs and adaptor proteins (VTI1, STX4, AP1B1, AP2A2, AP2S1, AP3D1 and AP4B1), which are required for fusion between vesicles carrying mature proteins and the cell surface, endosomes and lysosomes, are downregulated, predicting impairment in the delivery of proteins throughout the cell (5). Finally, inhibition of autophagy by the mTORC1 complex and downregulation of ATG1, which forms the phagophore assembly site (PAS) with ATG17 and ATG13 to initiate autophagy, indicates a decrease in the clearance of cellular debris which may result in cytosolic accumulations and contribute to motor neuron injury (6).

Microtubule-associated protein 1s (*MAP1S*) and 4 (*MAP4*), are involved in microtubule (MT) stabilisation [25], and are both downregulated (⇓2.8 and ⇓3, respectively), whereas the MT-destabilising protein stathmin (*STMN1*), is upregulated (⇑1.6) in the presence of mutant *CHMP2B*. Microtubule destabilisation may result in transport impairment, and this is supported by downregulation of several kinesin (*KIF1A* ⇓3, *KIF5C* ⇓2.8 and *KIF1C* ⇓2.2) and dynein transcripts (*DYNLRB1* ⇓2.8, *DYNLL2* ⇓3 and *DYNC1H1* ⇓3.5). In addition, both tubulin alpha and beta, main components of microtubules, are highly downregulated (*TUBA* ⇓4.5, *TUBB* ⇓5), probably in response to destabilising stimuli. The transcript for *Golgi apparatus protein 1* (*GLG1*), used as a marker to assess Golgi apparatus (GA) structure and function, is highly downregulated (⇓3.16) in *CHMP2B* motor neurons, as are other structural proteins: Golgi reassembly stacking protein 1 (*GORASP1* ⇓3.7) and components of the oligomeric Golgi complex 2 and 7 (*COG2* ⇓1.5 and *COG7* ⇓2.6).

Fusion of ER-derived vesicles with the Golgi requires the pairing of the v-SNARE, blocked early in transport 1 (*BET1*), with its t-SNARE complex comprising syntaxin 5 (*STX5*), Golgi SNAP receptor complex member 2 (*GOSR2*;⇑2.76) and *SEC22A*, *B* (⇑1.6), *C*. *SEC23* and *SEC24*, whose function is to coat the vesicles travelling along microtubules to the cis-Golgi, are also upregulated (⇑1.5 and ⇑1.8 respectively). In contrast, *USE1* (unconventional SNARE in the ER homologue 1), which is involved in retrograde transport from the Golgi to the ER [26], is downregulated (⇓2.66). Furthermore, two components of the coatomer protein complex I, *COPE* and *COPZ1* are downregulated (⇓3.8 and ⇓3 respectively). This complex is responsible for coating of the vesicles travelling between ER and Golgi. Also downregulated are the t-SNAREs, *VTI1* (⇓2.9) and *STX4* (⇓2.2), which encode proteins involved in vesicle docking and transport from the GA to the endosome. In addition, many of the adaptor-related protein complex transcripts are strongly downregulated, *AP1B1* (⇓2.29), *AP2A2* (⇓2.25), *AP2S1* (⇓3.35), *AP3D1* (⇓2.67) and *AP4B1* (⇓2), and these also play a key role in the transport of vesicles from the GA to the endosomal sorting pathway. *KIF1A*, *TUBB* and *MAP4* were selected for verification by Q-PCR and were found to be significantly downregulated by 2.96 (p = 0.0009), 3.07 (p<0.0001) and 3.52 fold (p = 0.0003), respectively (Table 5).

**Table 5:** Comparison of fold-changes calculated by microarray experiments and determined by Q-PCR.

Gene	Microarray fold change	Q-PCR fold change (p value)
MAP4	−3	−3.52 (p = 0.0003)
TUBB	−4.87	−3.07 (p<0.0001)
ATG1	−3.48	−2.2 (p = 0.003)
KIF1A	−3.02	−2.96 (p = 0.0009)
NCX1	3.21	25.8 (p<0.0001)
SERCA	2.12	1.66 (p = 0.0044)
E2F6	2.80	3.54 (p = 0.0112)
CDH13	5.66	10.43 (p = 0.0038)

The p value for the Q-PCR was calculated using an unpaired *t* test.

#### mTOR signalling and regulation of autophagy (Figure 3)

mTOR is a serine/threonine protein kinase that integrates the input from multiple upstream pathways, and whose activity is stimulated by insulin, growth factors, serum, phosphatidic acid, amino acids and oxidative stress [27], [28]. mTOR associates with several other proteins: Raptor, GβL and PRAS40, to form a complex known as mTORC1 [29]. mTOR is involved in many cellular processes, one of which is the inhibition of autophagy. Importantly, a key protein involved in autophagy activation, ATG1, is strongly downregulated (⇓3.48). ATG1 is essential for the formation of vesicles at the phagophore assembly site (PAS) [30], suggesting autophagy is impaired in CHMP2B motor neurons. In addition to its role in autophagy inhibition, the mTOR signalling pathway is involved in translation initiation. mTORC1 inhibits *PP2A* (protein phosphatase 2A), of which the regulatory subunit 4 is downregulated (⇓2.03). *PP2A* inhibits p70S6K, which binds to the eukaryotic translation initiation factor 3 (eIF3) when inactive [31]. Activation of p70S6K by mTORC1 causes it to release eIF3, allowing p70S6K to activate target proteins [31]. eIF3 consists of twelve non-identical subunits (eIF3 A(⇓2.03), B(⇓3.97), C(3.23⇓), D(⇓2.28), E, F, G(⇓1.80), H, I(⇓5.79), J, K and L) [32]. Upon release from p70S6K, eIF3 binds to ribosomal protein S6 (RPS6, ⇓2.28), which is part of the 40S ribosome subunit [33]. The eIF3/40S complex then forms a larger pre-initiation complex with eIF4E, eIF4G, eIF4B (⇓1.94) and eIFA (⇓3.04). eIF4A plays a role in resolving 5′UTR mRNA secondary structure, and thus allowing the ribosome to bind, and this helicase action is facilitated by eIF4B [34]. *ATG1* was selected for Q-PCR verification, and was downregulated 2.2 fold, p = 0.003 (Table 5).

#### MAPK signalling (Figure 4)

**Figure 4:**
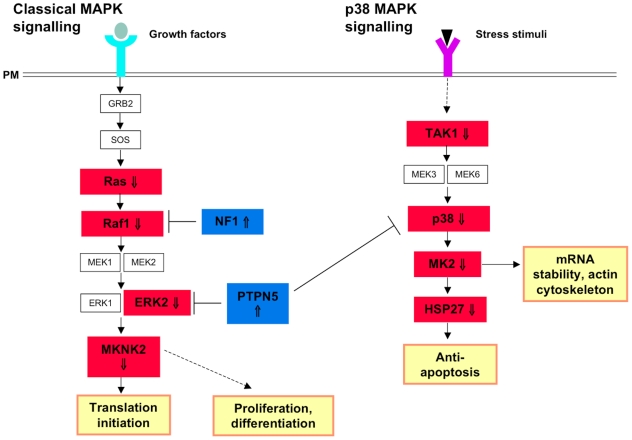
Defects in MAPK signalling as a result of mutations in *CHMP2B*. There are two main pathways through which MAP kinases signal: the classical MAPK pathway and the p38 MAP kinase pathway. In the classical signalling pathway, ligand binding results in receptor activation, which in turns leads to the activation of GRB2 and SOS. SOS catalyses the substitution of GDP for GTP on RAS (⇓), which then activates RAF1 (⇓). RAF1 subsequently phosphorylates MEK1/2, which in turn activate ERK1 and ERK2 (⇓). The ERK proteins activate MKNK2 (⇓), which is directly responsible for activation of proteins required for translation initiation. Downregulation of multiple core signalling components in combination with upregulation of the inhibitors, NF1 and PTPN5 predicts inability of the cell's basal response to growth factors. The p38 MAPK pathway is a signalling cascade that is distinct, but not exclusive from the classical MAPK signalling pathway. As with the classical MAPK pathway, multiple elements of the pathway are downregulated: TAK1(⇓), p38 (⇓), MK2 (⇓) and HSP27 (⇓) which may result in a decrease in mRNA stability and the cell's anti-apoptotic response. (PM = plasma membrane)

There are two main pathways through which MAP kinases signal: the classical MAPK pathway and the p38 MAP kinase pathway [35]. The first step in the activation of classical MAPK signalling is ligand binding to a receptor tyrosine kinase. The phosphorylated tyrosine of the target protein is bound by the SH2 domain of GRB2, which also contains an SH3 domain that binds to the proline-rich region of SOS. Once bound by GRB2, SOS catalyses the substitution of GDP to GTP on RAS (⇓4.96). GTP-bound RAS activates the MAPKKK, *RAF1* (⇓2.10), by phosphorylation. RAF1 subsequently phosphorylates the MAPKKs, MEK1/2, which in turn activate the MAP kinases *ERK1* and *ERK2* (⇓1.71). MAPK interacting serine/threonine 2 (MKNK2) is directly activated by ERK, and is itself downregulated (⇓3.24). MKNK2 contributes to the basal phosphorylation of eIF4A, which is essential for translation initiation [36]. Downregulation of these core pathway components suggests repression of the basal response to growth factors, an effect that appears to be amplified by upregulation of the Ras inhibitor, NF1 (⇑1.81) and the ERK1/2 inhibitor, PTPN5, (⇑1.59).

Activation of p38 is mediated by multiple upstream kinases and this pathway plays a key role in the cell's stress response as well as the phosphorylation of multiple target proteins including phospholipase A_2_ and MAP tau. TAK1 (⇓2.42) is an upstream kinase that phosphorylates MAPK kinases 3 and 6 (MEK3, 6), which subsequently phosphorylate p38 (⇓5.34). There is strong downregulation of the p38 MAPK signalling pathway, which is strengthened by upregulation of the p38 (and ERK1/2) inhibitor PTPN5 (⇑1.59). Additionally, the p38 target, MK2 is downregulated (⇓1.60), which plays a role in the regulation of mRNA stability and the reorganisation of actin [35]. MK2 activates Hsp27 (⇓12.85), which binds to and inactivates the pro-apoptotic molecules caspase-3, caspase-9 and cytochrome *c* [37].

#### Calcium signalling (Figure 5)

**Figure 5:**
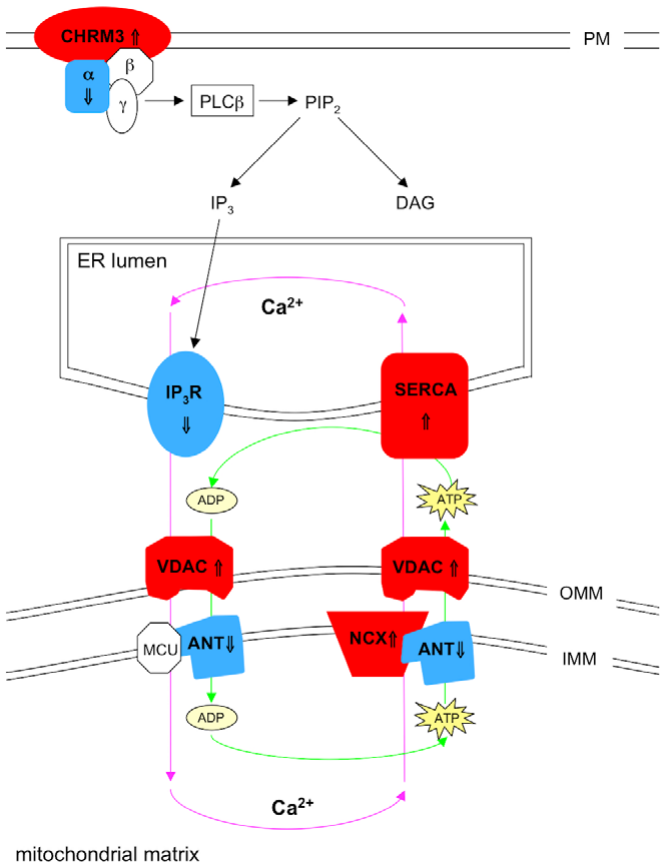
Defects in calcium signalling as a result of CHMP2B mutations. Downregulation of the α-subunits, G_q_ (⇓) and G_q11_ (⇓) indicates a decrease in PLCβ activation, despite upregulation of the cholinergic receptor CHRM3 (⇑). A decrease in PLCβ activity would reduce the amount of phosphatidylinositol 4,5-bisphosphate (PIP_2_) hydrolysed to inositol 1,4,5-triphosphate (IP_3_) and diacylglycerol (DAG). Combining the decrease in IP3, with downregulation of its receptor, IP3R (⇓), would reduce the opening of a Ca^2+^-release channel on the ER membrane, which allows Ca^2+^ to exit the ER lumen into the cytosol. SERCA, which pumps Ca^2+^ out of the cytoplasm into the ER lumen is upregulated, so these findings predict an increased Ca^2+^ concentration in the ER lumen. Upregulation of NCX1(⇑) and VDAC3(⇑), and downregulation of ANT3(⇓), predict that mitochondria are increasing the amount of calcium pumped out of the matrix, but decreasing the amount of ATP leaving the organelle. Amplifying the aberrant intracellular calcium levels, in addition to reducing the amount of energy available for cellular processes. (PM = plasma membrane; OMM = outer mitochondrial matrix; IMM = inner mitochondrial matrix).

The G protein coupled receptor, cholinergic receptor, muscarinic 3 (*CHRM3*), is upregulated (⇑2.92) in *CHMP2B* mutant motor neurons. Muscarinic_3_ (M_3_) receptors couple to phospholipase Cβ (PLCβ) via the G_q_ class α-subunits [38]. However, as both *G_q_* and *G_q11_* are downregulated (⇓3.08 and ⇓2.51, repectively), this would predict a decrease in PLCβ activation, despite the increase in *CHRM3* transcription. PLCβ functions by catalysing the hydrolysis of phosphatidylinositol 4,5-bisphosphate (PIP_2_) to inositol 1,4,5-triphosphate (IP_3_) and diacylglycerol (DAG). IP_3_ binds to the IP_3_ receptor (*IP_3_R*, ⇓1.93), which is located on the ER membrane, resulting in the opening of a IP_3_-gated Ca^2+^-release channel, thus allowing calcium to exit the ER lumen and enter the cytoplasm, where it can propagate the signal by activating protein kinase C (PKC). Following depletion of ER stores, calcium is re-accumulated through sarco-endoplasmic Ca^2+^ ATPase (*SERCA*, ⇑2.12), which is directly controlled by the ATP supplied by mitochondria [39]. ANT3 catalyses the exchange of ATP for ADP from the mitochondrial matrix through the inner mitochondrial membrane into the inter-membrane space, and is downregulated (⇓1.82). Solute carrier family 8, sodium/calcium exchanger 1 (*NCX1*, ⇑3.21) and voltage-dependent anion channel 3 (*VDAC3*, ⇑2.93), are located on the inner mitochondrial membrane and outer mitochondrial membrane, respectively, and pump calcium out of the mitochondrial matrix and into the cytoplasm, where it is transported to the ER lumen via *SERCA* [39]. *NCX1* and *SERCA* were selected for verification by Q-PCR, and were found to be upregulated by 25.75 fold (p<0.0001) and 1.66 fold (p = 0.0044), respectively (Table 5).

We additionally validated E2F transcription factor (*E2F6*) and cadherin 13 (*CDH13*) by Q-PCR. These genes had microarray fold changes of 2.80 and 5.66, respectively, and Q-PCR fold changes of 3.54 (p = 0.0112) and 10.43 (p = 0.0038), respectively (Table 5).

### Cellular pathology of CHMP2B mutations

**Figure 6:**
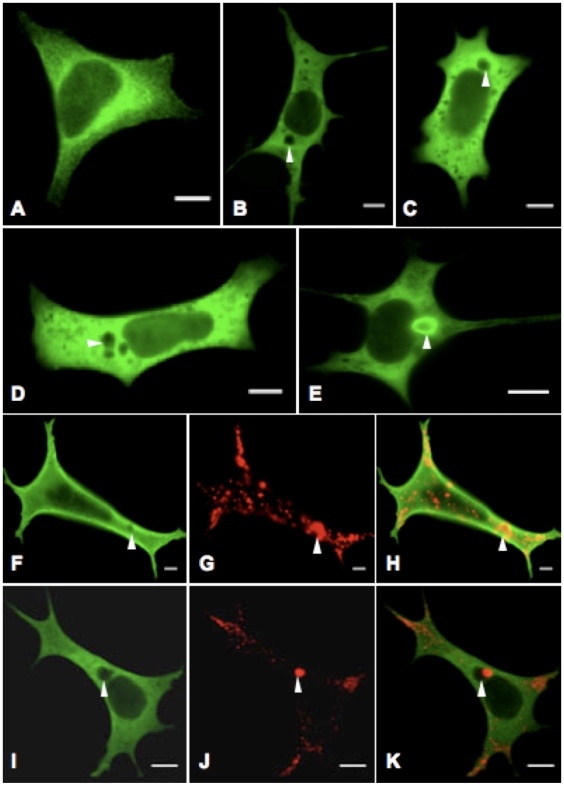
Overexpression of mutant CHMP2B produces an aberrant phenotype in HEK-293 cells. Cells were transfected with vectors encoding recombinant protein c-Myc-CHMP2B with either the wild-type or I29V, T104N or Q206H mutant sequence, and stained with FITC-conjugated antibody to c-Myc. Transfection with wild-type CHMP2B (A) results in generalised cytoplasmic expression, whereas the mutant isoforms I29V (B), T104N (C) and Q206H (D) resulted in cytoplasmic vacuoles of varying size (indicated by arrowheads). Another striking observation was the presence within cells expression mutant CHMP2B of circular CHMP2B accumulations in the cytoplasm, termed halos (E). Cells were doubly stained with antibodies to c-Myc (F & I), as well as CD63 (G & J), and merged to show co-localisation (H & K). CD63 co-localises with the small vacuoles found in cells transfected with WT CHMP2B (F–H). However, CD63 staining does not co-localise with large vacuoles in mutant expressing cells (cells transfected with T104N shown), but are found on the vacuole edge (I–K). Images were taken on a Zeiss LSM 510 confocal microscope, ×63 obj. Bar, 10µm.

**Figure 7:**
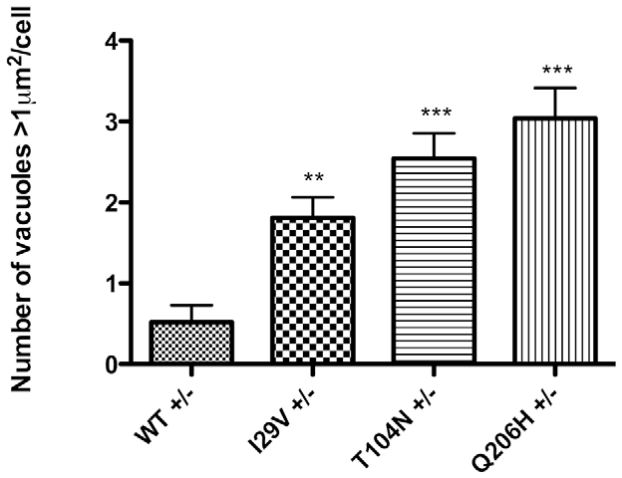
Mutant CHMP2B causes large cytoplasmic vacuoles. Cells expressing 3 different mutant CHMP2B had significantly more vacuoles with an area greater than 1µm^2^ in than cells expressing wild-type protein (One-way ANOVA p<0.0001, +/−: cells grown in DMEM with FCS, without antibiotic).

**Figure 8:**
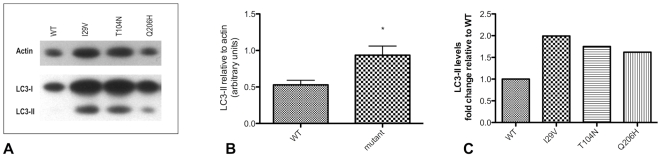
LC3-II levels are increased in cells expressing mutant CHMP2B. A representative image of western blotting to LC3 and actin (A), showing increased levels of LC3-II in COS-7 cells expressing mutant CHMP2B protein (lanes 2–4) compared to cells expressing wild-type protein (lane 1). LC3-II levels relative to an actin loading control were measured using densitometry (B), and this showed increased levels of LC3-II in mutant expressing cells compared to WT (mean ±S.E.M., n = 3) (Mann Whitney p = 0.0242). Average fold changes in LC3-II levels normalised to WT are shown for each of the three mutants (C).

To identify pathogenic effects of *CHMP2B* mutations, HEK-293 cells were transfected with constructs containing myc-tagged full-length *CHMP2B* with either wild-type or p.I29V, p.T104N or p.Q206H mutant *CHMP2B*. 24 hours after transfection, cells expressing wild-type CHMP2B showed a typically diffuse pattern of staining throughout the cell (Figure 6A). Cells expressing mutant CHMP2B isoforms had large cytoplasmic vacuoles that were devoid of lumenal staining for the recombinant protein, such vacuoles were rarely seen in cells expressing wild-type protein (Figure 6B–D). Image analysis showed there were significantly more vacuoles with an area greater than 1µm^2^ in cells expressing mutant CHMP2B protein compared to wild-type (one way ANOVA: p<0.0001, Figure 7). Additionally, the presence of mutant CHMP2B increased the number of cells with vacuoles greater than 1µm^2^ in area (Bonferroni post-test, p<0.001), as did serum withdrawal (Bonferroni post-test, p<0.0001). A second phenotype observed in the mutant cells was the presence of vacuoles with an accumulation of CHMP2B mutant protein on the outer membrane, termed halos (Figure 6E). The number of cells with halos in cells expressing the p.T104N isoform of *CHMP2B* was significantly increased (Bonferroni post-test, p<0.05). When cells were deprived of serum the presence of halos was even more striking in mutant cells. Co-staining with CD63, a tetraspanin that is abundant in late endosomes and lysosomes, revealed an interesting change in staining pattern in cells expressing mutant *CHMP2B*. In cells expressing WT CHMP2B, CD63 co-localises with small vacuoles within the cytoplasm (Figure 6F–H). In cells expressing mutant CHMP2B, CD63 does not co-localise with the large cytoplasmic vacuoles caused by mutant CHMP2B transfection, but instead is found on the membrane of these aberrant structures (Figure 6 I–K). Immunoblotting of LC3 typically reveals two bands, LC3-I (18kDa) and LC3-II (16kDa). During the formation of autophagosomes, the cytoplasmic form of LC3 (LC3-I) is recruited, where it undergoes site-specific proteolysis and lipidation, generating LC3-II which sequesters to the membrane of the autophagosomes [40]. The level of LC3-II can be used to monitor autophagic activity, as it correlates with the number of autophagosomes. Western blotting for LC3 in COS-7 cells showed a significant increase in LC3-II levels in cells expressing mutant CHMP2B compared to those expressing wild-type protein (Figure 8).

## Discussion

In our cohort, we have identified mutations in *CHMP2B* in four out of 433 individuals with ALS, giving a frequency of just less than 1%. This is approximately half of the frequency of SOD1 mutations, which have been reported to account for approximately 20% of familial ALS cases [41], and 2% of all ALS cases [42], [43], [44]. Of note, only 1 of our 4 cases of *CHMP2B* associated ALS/MND had a discernible family history compatible with ALS in a second-degree relative. This supports the hypothesis that some apparently sporadic ALS cases have a genetic component. Evidence from a UK twin study, which examined concordance in both mono- and dizygotic twins (having first excluded probands from families in which dominant inheritance of MND had already been identified), estimated the heritability of ALS to be between 0.38 and 0.85; indicating that genetic factors are likely to make a substantial contribution to the sporadic form of the disease [45]. Although the present study has not been able to document the segregation of *CHMP2B* in multiple affected members of specific pedigrees in ALS, we believe our results support the body of evidence for the contribution of genetic factors to apparently sporadic ALS.

All 4 cases in this report were negative for changes in *SOD1*, *ANG*, *TDP43*, *VAPB and FUS/TLS*. Clinically, all four cases presented with a phenotype consistent with a lower motor neuron phenotype of ALS. In this cohort only 40 cases are recorded clinically as having a lower motor neuron predominant phenotype and *CHMP2B* mutations were found in 4 (10%) of these cases. In our autopsy cohort, 15/123 cases had a LMN phenotype pathologically. Of these 15, three lack any evidence of ubiquitin/TDP43 neuronal inclusion pathology, one in the presence of a mitochondrial transfer RNA gene mutation [46], and another as one of a pair of brothers with MND and colonic neoplasia recently found to have a mutation in *FUS/TLS* [47]. As such *CHMP2B*-related ALS is highly over-represented in this LMN predominant clinical subgroup. It is interesting to note that although mutations in *CHMP2B* were originally identified in patients with FTD, none of the four cases we identified had clinically apparent cognitive changes and there were no noteworthy pathological changes in the hippocampus.

A previous report of 166 familial and 372 sporadic “classical” ALS cases of predominantly Anglo-Celtic origin from Australia and London found no evidence of mutations in *CHMP2B* [48]. However, population differences in other ALS pathogenic mutations have been previously reported as exemplified by the lack of *TARDBP* mutations in some populations and [49], [50] the very low frequency of *SOD1* mutations reported in some countries such as the Netherlands [51].

In silico analysis of the identified amino acid substitutions predicts that the p.I29V and p.Q206H mutations decrease protein stability (http://gpcr2.biocomp.unibo.it/cgi/predictors/I-Mutant2.0/I-Mutant2.0.cgi). The native threonine of the p.T104N change is predicted to be a site of phosphorylation (www.cbs.dtu.dk/services/NetPhos), thus substitution of threonine with asparagine is predicted to affect protein activity. We did not identify any of the described codon changes in our 500 controls, whilst previous studies have sequenced 1495 samples for exon 3 and 2035 samples for exon 6, without identifying the p.T104N and p.Q206H substitutions [3], [4], [48], [52]. We therefore propose that these changes are not rare benign polymorphisms, but are associated with disease. Although the p.I29V substitution has been reported in a single control sample, as well as in a familial FTLD case [53], screening of our 500 controls, 640 previously screened controls, 708 ALS cases and 546 FTD cases [3], [4], [48], [52] has failed to detect this change. Therefore, whilst it is recognised that not all missense mutations are pathogenic [54], the clinical, neuropathological and cellular phenotype common to all 3 CHMP2B mutations, and which is distinct from controls, supports the proposal that all 3 nucleotide substitutions described in this report are associated with a lower motor neuron dominant-ALS. The c.-151C>A polymorphism is predicted to alter the binding site for an unknown transcription factor (www-bimas.cit.nih.gov/molbio/index.shtml). However, there is no significant difference in frequency between subjects and controls (Chi Square p = 0.9), and therefore this change is likely to represent a non-functional polymorphism.

The pathology is rather stereotypical and, whilst firmly within the ALS/MND spectrum, appears to represent a rather distinctive lower motor neuron variant. The inclusion morphology does not correspond to the predominant pattern seen in classical ALS/MND, where skein inclusions predominate in ∼90% of cases, so that the absence of classical skeins in this group is distinctive. Bunina bodies are found in up to 75–80% of ALS/MND cases, and their absence in these 4 patients is again suggestive of an atypical group. While these features are not sufficiently distinctive to allow a morphological prediction of *CHMP2B*-related MND, our results indicate that a predominance of compact inclusions in a PMA case may warrant examination of the *CHMP2B* gene.

Microarray analysis of the gene expression profile of motor neurons with *CHMP2B* mutations compared to neurologically normal control samples reveals some interesting changes to genes involved in key cellular processes, many of which are distinct to those shown by motor neurons isolated from *SOD1*-related ALS cases (Kirby et al, manuscript under review). *CHMP2B* cases show downregulation in multiple transcripts encoding proteins involved in the transport of cargoes along microtubules, suggesting impairments in axonal transport (*KIF1A*, *KIF1C*, *KIF5C DYNLRB1*, *DYNLL2* and *DYNC1H1*), a phenomenon that has been well documented in multiple neurodegenerative diseases, including a SOD1-mediated model of ALS [55]. Interestingly, the dysregulation of microtubule proteins *MAP1S*, *MAP4* [25] and stathmin, predicts loss of cell structure and transport network. It has been shown that stathmin overexpression in HeLa cells leads to Golgi fragmentation and microtubule disassembly, which are the same events observed in transgenic G93A SOD1 mice [56]. Stathmin overexpression and Golgi fragmentation seem to be early events in the neurodegenerative cascade characteristic of ALS and other neurological diseases [57], and has been confirmed by transcriptional analysis in pre-symptomatic G93A SOD1 mice [19]. *ATG1* (3.48 fold) is strongly downregulated, and is part of a complex with *ATG17* and *ATG13*. This complex is required for the formation of vesicles at the phagophore assembly site, which is a crucial step for autophagy initiation [30]. A decrease in autophagy initiation may result in the accumulation of cytosolic aggregates, which could potentially contribute to motor neuron injury. It is of great interest that a recent study in a fly model of Huntington's disease combined the treatment of rapamycin and lithium [58]. Inhibition of mTOR by rapamycin and the induction of mTOR-independent autophagy by lithium resulted in an increase in autophagy in both an mTOR-dependent and mTOR-independent manner, enhanced the clearance of protein aggregates and provided greater protection than single treatment with either of these molecules [58]. In addition, our results predict a global repression of protein translation within the cell due to the downregulation of multiple translation initiation factors. Translation initiation is a critical checkpoint and step of the translation process, and is the stage at which control of mRNA translation is most strongly exerted [59]. This may be an effect of end-stage disease, where the cell is attempting to survive at the expense of general protein synthesis, or it may be in response to a compromised ER trying to prevent accumulation of misfolded proteins. The classical MAPK cascade and the p38 signalling pathway both show downregulation of multiple components, alongside upregulation of inhibitors of signal amplification. This would predict that the cell is unable to respond normally to stimulation from growth factors, cytokines or environmental stresses. Downregulation of *HSP27*, which plays a role in neuronal survival, in conjunction with the altered transcriptional levels of *MKNK2* and *AKT* suggests that the motor neurons are in a state that predisposes to apoptosis. Interestingly, pharmacological abrogation of p38 by SB203580, resulted in the accumulation of large autolysosomes [60]. Downregulation of p38 is therefore potentially very important as *CHMP2B* functions in degradation and recycling pathways, and mutations result in autophagosome accumulation [12]. The downregulation of *Gα_q_* and *Gα_11_* suggests a reduction in IP_3_ levels as a result of decreased PLCβ activity, and this in conjunction with the downregulation of IP_3_R, predicts a reduction in calcium release from the ER. In addition, the upregulation of *SERCA* and the mitochondrial calcium transporters *NCX1* and *VDAC3*, may result in excess calcium being transported out of the mitochondria and into the ER lumen, having the net effect of an increased [Ca^2+^] in the ER lumen and reduced [Ca^2+^] in the mitochondria. Tight control of ER Ca^2+^ stores is crucial for cell survival, and it has been previously demonstrated that increased ER calcium levels trigger ceramide-induced apoptosis [61].

We have demonstrated in a HEK-293 cellular model that transfection of constructs carrying p.I29V, p.T104N or p.Q206H *CHMP2B* mutations results in the formation of large cytoplasmic vacuoles (Figure 6 B–D). Induction of autophagy resulted in an increase in the number of vacuoles, providing further evidence that functional CHMP2B is required for the degradation of cytoplasmic components. These mutations caused aberrant CD63 accumulation within the cytosol (p.T104N: Figure 6 I–J; p.I29V & p.Q206H: data not shown). CD63 is found abundantly in lysosomes, and its trafficking and cellular distribution is tightly regulated [62]. The small vacuoles found in cells expressing WT CHMP2B co-localised with large areas of CD63 staining – displaying the normal physiological fusion of late endosomes with lysosomes (Figure 6 F–H). In contrast, cells expressing mutant CHMP2B often showed CD63 immunostaining on the membranes of large vacuoles (Figure 6 I–K). These data indicate that the missense *CHMP2B* mutations identified in our patient cohort disrupt the fusion of the lysosome with the large cytoplasmic vacuoles, suggesting that mutant CHMP2B is altering the dynamics of this fundamental process. This hypothesis is further supported by the transfection of FTD-related *CHMP2B* truncating mutations in neuroblastoma cell lines, which led to the formation of large vesicles, with a morphology in keeping with aberrant endosomes [52].

The MVB pathway has an essential role in the delivery of ubiquitinated proteins to the lysosome for degradation, and is highly conserved from yeast to humans. It has been demonstrated that mice deficient in genes essential for autophagy are unable to effectively clear cellular debris, leading to the accumulation of polyubiquitinated proteins within inclusion bodies and subsequent neurodegeneration [63], [64]. Additionally, there is strong evidence that *CHMP2B* mutations result in neuronal death; dendritic retraction and cell death seen in cortical neuron cultures transfected with CHMP2B^Intron5^ is a result of the failure of the aberrant protein to dissociate from the other subunits of the ESCRTIII prior to the next round of sorting, a step critical for the formation of MVBs [12]. Transfection of HeLa cells with CHMP2B^Intron5^ and CHMP2B^Δ10^ results in increased levels of ubiquitin and p62 and inhibition of protein degradation through the process of autophagy [13]. Inhibiting autophagy induction in cells transfected with mutant CHMP2B resulted in delayed neuronal loss, further implicating autophagy in the process of neurodegeneration [65], [66]. In the present study, we have demonstrated that LC3-II levels are significantly increased in cells expressing mutant CHMP2B. Autophagosomes sequester the target organelle/protein and fuse with lysosomes, where the contents – and the LC3-II present on the luminal side of the autophagosome – are degraded [67]. The half-life of LC3-II is short due to the balance of autophagosome synthesis and degradation; thus the increased levels of LC3-II in mutant-expressing cells is indicative of an alteration in the degradation of cargo by the autophagic pathway. We therefore propose that the *CHMP2B* mutations we have identified may contribute to motor neuron injury through dysfunction of the autophagic clearance of cellular proteins, and the formation of ubiquitin positive inclusions with compact morphology (Figure 2). Further mutation screening now needs to take place in cohorts of patients, particularly those with a lower motor neuron disease phenotype, and from different geographical areas. Identification of *CHMP2B* mutations showing segregation with familial disease would establish conclusively the role of *CHMP2B* as a causative gene in ALS.

## Supporting Information

Table S1 The 891 genes downregulated and 561 genes upregulated in motor neurons with CHMP2B mutations, grouped into biological processes.
(0.23 MB XLS)

Figure S1 Multiple protein alignment of human CHMP2B, mutant isoforms and orthologues. Amino acids are shaded based on their properties; polar amino acids are bright, whereas non-polar residues are darker. Missense mutations identified in the MND cohort (p.I29V, p.T104N and p.Q206H) are labelled *.
(5.73 MB TIF)
