# Supplementary figures and images for: Mutations in CHMP2B in Lower Motor Neuron Predominant Amyotrophic Lateral Sclerosis (ALS)
Source: PLoS One. 2010 Mar 24;5(3):e9872. doi: 10.1371/journal.pone.0009872 (PMC2844426; doi:10.1371/journal.pone.0009872)

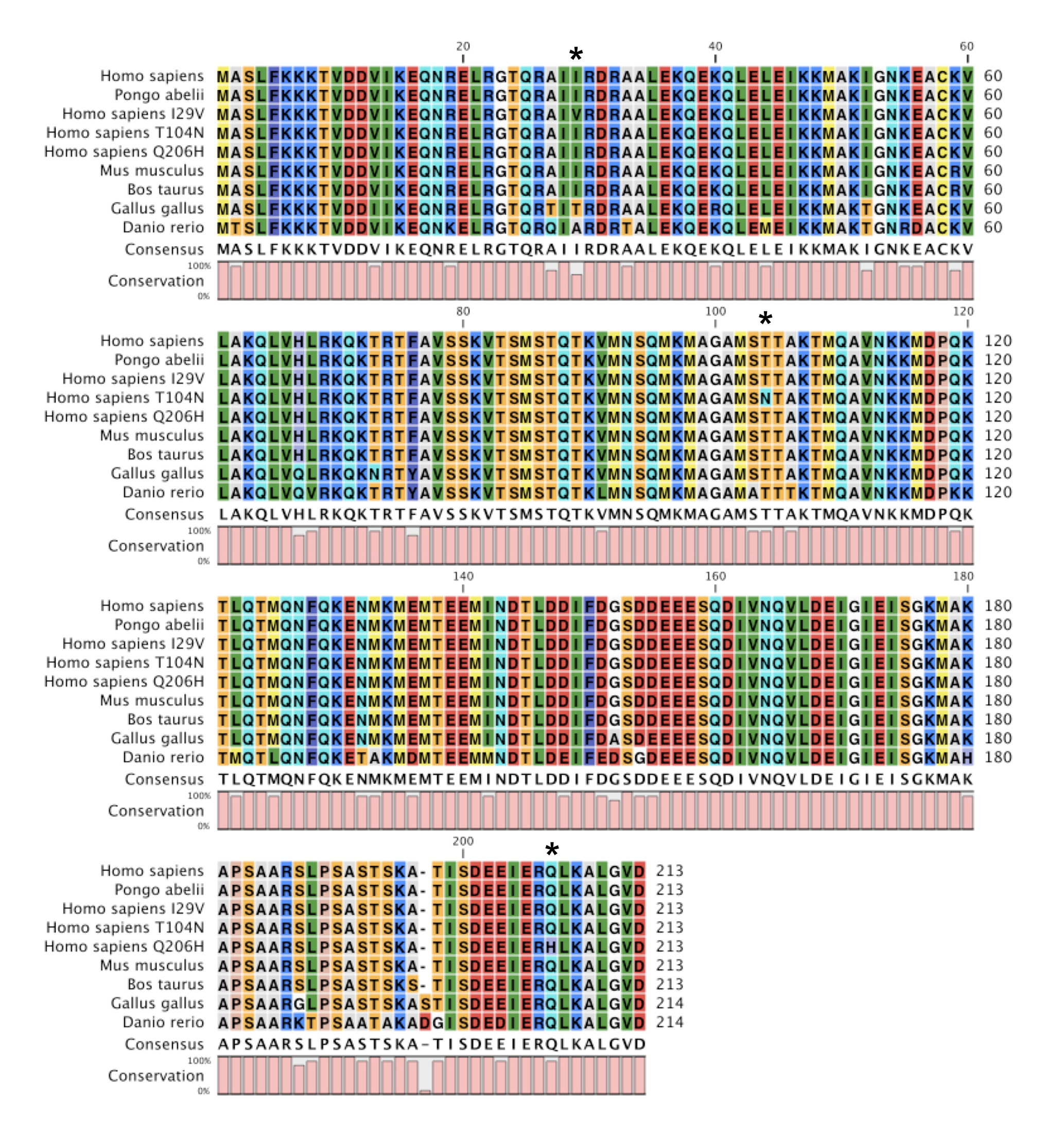

Supplement: Figure S1 — Multiple protein alignment of human CHMP2B, mutant isoforms and orthologues. Amino acids are shaded based on their properties; polar amino acids are bright, whereas non-polar residues are darker. Missense mutations identified in the MND cohort (p.I29V, p.T104N and p.Q206H) are labelled *. (5.73 MB TIF) [file pone.0009872.s002.tif]
